# A systematic review, umbrella review, and quality assessment on clinical translation of stem cell therapy for knee osteoarthritis: Are we there yet?

**DOI:** 10.1186/s13287-023-03332-5

**Published:** 2023-04-15

**Authors:** Zhizhong Shang, Pingping Wanyan, Baolin Zhang, Mingchuan Wang, Xin Wang

**Affiliations:** 1 The First Clinical Medical College of Lanzhou University, Lanzhou, 730000 China; 2 Gansu University of Chinese Medicine, Lanzhou, 730000 China; 3 The Second Hospital of Lanzhou University, Lanzhou, 730000 China; 4 Chengren Institute of Traditional Chinese Medicine, Lanzhou, 730000 Gansu Province China; 5 Department of Spine, Changzheng Hospital, Naval Medical University, Shanghai, 200003 China

**Keywords:** Knee osteoarthritis, Stem cells, Adverse reactions, Efficacy, Evidence quality, Systematic review

## Abstract

**Background:**

The success of stem cell therapy for knee osteoarthritis (KOA) in preclinical animal models has accelerated the pace of clinical translation. However, it remains uncertain whether the current scientific evidence supports the clinical application of stem cells in treating KOA. A comprehensive evaluation of the safety and efficacy of stem cell therapies and scientific evidence quality is necessary.

**Methods:**

Using “stem cells” and “knee osteoarthritis” as the search terms, several databases, including PubMed, Web of Science, Cochrane, Embase, and Clinicaltrials.gov, were searched on August 25, 2022, and updated on February 27, 2023. Clinical studies that reported adverse reactions (ARs) of stem cell therapy in KOA patients were included without limiting the type of studies. Quantitative systematic reviews of stem cell therapy for KOA that conducted meta-analysis were included. Two researchers conducted literature screening and data extraction independently, and the evidence quality was evaluated according to the Institute of Health Economics and AMSTAR 2 criteria.

**Results:**

Fifty clinical studies and 13 systematic reviews/meta-analyses (SRs/MAs) were included. Nineteen ARs were reported in 50 studies, including five knee-related ARs, seven common ARs, and seven other ARs. Some studies reported over 10% prevalence of knee pain (24.5%; 95% CI [14.7%, 35.7%]), knee effusion (12.5%; 95% CI [4.8%, 22.5%]), and knee swelling (11.9%; 95% CI [3.5%, 23.5%]). Additionally, two studies have reported cases of prostate cancer and breast tumors, respectively. However, these two studies suggest that stem cell therapy does not bring significant ARs to patients. SRs/MAs results revealed that stem cell therapy relieved pain in patients over time but did not improve knee function. However, current clinical studies have limited evidence regarding study objectives, test designs, and patient populations. Similarly, SRs/MAs have inadequate evidence regarding study design, risk of bias assessment, outcome description, comprehensive discussion, and potential conflicts of interest.

**Conclusions:**

The inefficacy of stem cells, the risk of potential complications, and the limited quality of evidence from current studies precluded any recommendation for using stem cell products in patients with KOA. Clinical translation of stem cell therapies remains baseless and should be cautiously approached until more robust evidence is available.

*PROSPERO registration number*: CRD42022355875.

**Supplementary Information:**

The online version contains supplementary material available at 10.1186/s13287-023-03332-5.

## Background

The global prevalence of knee osteoarthritis (KOA) increases with age, with 16% of people over 15 years old and 22.9% of people over 40 years old [1]. Aggravating pathophysiological changes, including articular cartilage destruction, subchondral bone sclerosis, cystic degeneration, and osteophyte formation, cause knee joint pain and loss of function, making KOA difficult to treat [2]. Most KOA patients seek treatment when they experience pain, swelling, or limited mobility in the knee joint [3]. KOA management typically involves a comprehensive approach to symptom relief, including physical therapy, drug therapy, and surgical intervention [4, 5]. Drug therapy, such as topical or oral non-steroidal anti-inflammatory drugs (NSAIDs) and intra-articular corticosteroid injections, is a key treatment option for KOA [6]. While NSAIDs are the first-line treatment for KOA, they have several toxicities, including gastrointestinal irritation and ulceration, bleeding, and decreased renal blood flow in azotemia [7]. Therefore, KOA patients taking anticoagulants may benefit from NSAIDs such as COX-2 inhibitors, particularly celecoxib, which does not increase bleeding risk. Patients with dyspepsia should use a proton pump or COX-2 inhibitors, while those with a history of peptic ulcer bleeding should avoid NSAIDs altogether [7]. Patients who cannot tolerate NSAIDs or fail to respond to this treatment may consider corticosteroid injections as an alternative option [8]. Moreover, recently developed biological agents such as *trans*-capsaicin injection and lutikizumab inhibit inflammatory factors, including Interleukin (IL)-1α, IL-1β, and TNF-α; however, they have limited therapeutic effects [9–11]. Knee replacement as an effective treatment for the advanced disease has the risk of multiple complications, a high cost, and a second revision [12]. Therefore, novel effective treatments must be explored.

With the development of tissue engineering and regenerative medicine, stem cells have emerged as a prominent area of research due to their remarkable ability to proliferate and differentiate in multiple directions, thereby promoting cartilage formation, vascularization, and anti-inflammatory and immunomodulatory effects [13]. Stem cell therapy has demonstrated significant progress in preclinical animal models of KOA and has been successfully applied in other diseases such as hematological malignancies, burns, and corneal transplants, garnering attention from patients, clinicians, pharmaceutical companies, and the media [14–16]. However, stem cells have been integrated into clinical practice primarily due to media hype rather than solid scientific evidence [17, 18]. Since KOA is not a life-threatening disease, the acceptable risk level of KOA will be lower than that of life-threatening diseases. Thus, the benefits and risks of stem cell therapy for KOA patients remain uncertain, and many qualitative and subjective clinical trials have failed to provide reliable answers [19].

Currently, the explosive growth of preclinical and clinical research, while rapidly advancing the clinical translation of stem cell therapy, may weaken or even ignore other more important issues [20]. It consists primarily of the following four aspects. (1) Stem cell therapy depends on its safety. Stem cell injections may cause cell transformation or premature cell differentiation [21]. Although adverse reactions associated with stem cell therapy have not been reported, the immunomodulatory function of stem cells may be involved in tumor development and occurrence [22]. (2) The effectiveness of stem cell therapy is controversial. The SRs/MAs of 13 randomized controlled trials (RCTs) revealed a statistically insignificant difference between intra-articular injections of mesenchymal stem/stromal cells (MSCs) and placebo in improving knee function and pain in patients [23]. Similarly, SRs/MAs of five RCTs also revealed stem cell transplantation did not repair cartilage damage or improve knee function in patients [24]. (3) Clinical studies have a poor quality of evidence. Although some studies claim to have found positive results, we must consider the quality of the studies. To date, only case reports, small cohort studies, and RCTs of low quality have been published [25]. The limited number of patients, absence of randomization and blinding, and presence of confounding factors all contribute to the potential bias [26] and do not demonstrate the validity of stem cells [27]. (4) The issues of stem cell origin, manipulation, transformation, and regulation have not been well addressed. For example, the ethical controversy of embryonic stem cells, limitations in the differentiation potential of adult stem cells, altered properties of stem cells following in vitro culture [28], the risk of infection transmission during stem cell transplantation [29], and variations in the quality of stem cell preparations from different suppliers [30]. Additionally, there are regulatory controversies surrounding stem cell therapy in different countries. For instance, the U.S. Food and Drug Administration has established a strict three-level regulatory system, “regulations-regulation-guidance principles”, for stem cell research, whereas Japan's regulations are relatively loose and open, allowing stem cell products to receive conditional approval with only ten patients with positive clinical data. Other countries, including China and India, face challenges in supervising stem cells and have not implemented a classified supervision system for the characteristics and applications of stem cells [31].

Clinical trials are well ahead of the available scientific evidence, but we must prioritize the health and welfare of patients over material gain because no one can afford serious consequences, including the potential for stem cell therapy to promote tumor formation [32]. Since several SRs/MAs have been conducted to summarize the efficacy of stem cells, duplicate validity evaluations are unnecessary. However, the current results of SRs/MAs are inconsistent, and the evidence quality is limited [23, 33–35]. Published SRs/MAs have not primarily focused on the safety of stem cell therapies. That’s why this study will assess clinical trials based on stem cell safety and SRs/MAs-based stem cells efficacy and evidence quality from current studies to clarify the feasibility of clinical translation of stem cell therapies.

## Methods

### Guideline and protocol

This systematic review and meta-analysis followed the guidelines of the Preferred Reporting Items for Systematic Reviews and Meta-Analyses (PRISMA) [36]. The review protocol has been registered with PROSPERO, number CRD42022355875 (https://www.crd.york.ac.uk/prospero/).

### Inclusion and exclusion criteria

Eligible studies met the criteria of the PICOS (participants, interventions, comparators, outcomes, and study design) [37].

#### Systematic review

*Inclusion criteria* (1) P: Knee osteoarthritis (KOA). (2) I: No restrictions on stem cell source, type, or transplant dose. (3) C: No restrictions on control groups. (4) O: Safety outcomes [mainly refer to the incidence of adverse reactions (ARs)]. (5) S: Descriptive studies, analytical studies, and experimental studies.

*Exclusion criteria* Studies of cartilage defects and their repair; studies on non-stem cell therapies like platelets, plasma, bone marrow aspirates, and stromal vascular fractions; studies reporting only the effectiveness of stem cells; studies that do not fit the research type, including reviews, conference abstracts, letters; studies with inaccessible or insufficient data.

#### Umbrella review

(1) Stem cell therapy must have a demonstrably superior therapeutic effect compared to conventional therapies, such as symptomatic therapy or placebo. Thus, we only considered SRs/MAs that make this comparison. (2) We analyzed several different knee function scales, including the Knee Osteoarthritis Outcome Score (KOOS), Western Ontario and McMaster Universities Osteoarthritis Index (WOMAC), and International Knee Documentation Committee (IKCD), and others. However, given the similarity of these scales and the recommended level, we chose to focus on the WOMAC and Visual Analog Scale (VAS) in our analysis [38]. (3) We only included systematic reviews with meta-analyses, generally considered more quantitative and reliable than qualitative reviews [39]. (4) We excluded SRs/MAs that combined data from different time points for analysis because of the large variation in treatment effects of stem cells at different follow-up times.

### Data sources and searches

Candidate studies were identified through searches of PubMed, Web of Science, Cochrane, Embase databases, and Clinicaltrials.gov from their inception to August 25, 2022, and an update retrieval on February 27, 2023. We also combed the reference lists of identified articles for additional relevant publications. Search terms were as follows: (stem cell OR stem cells) and (knee osteoarthritis, or knee and osteoarthritis). The detailed search strategies are provided in Additional file 1: Table S1 of Appendix 1. We did not conduct a separate search for SRs/MAs because they were included in the above search results.

### Literature screening and data extraction

Two independent evaluators screened all papers based on the inclusion and exclusion criteria. Any discrepancies in their assessments were resolved through the involvement of a third party, and missing information was obtained by contacting the authors. We sent up to three e-mails to the corresponding author of each paper to request missing materials. Otherwise, we considered our request to be unanswered. The initial screening process involved reviewing the title and abstract of each paper to eliminate irrelevant studies. After this initial screening, the full text of the remaining papers was carefully read to determine final inclusion. Data extraction included (1) Clinical trials: authors, country, year, study type, a sample size of a stem cell group, age, gender, Kellgren–Lawrence (K–L) grade, type of stem cells, source, transplantation dose, and follow-up time. (2) SRs/MAs: authors, year, type of study included, number of studies, the total number of participants, type of stem cells, transplantation dose, databases searched, PRISMA guidelines, quality assessment tools, and outcome indicators of effectiveness and safety.

### Quality assessment

To assess safety in clinical trials, we only extracted data on ARs in the stem cell group, equivalent to conducting an uncontrolled single-arm study or a case series. To assess the evidence quality of the included studies, we used the quality evaluation tool developed by the Canadian Institute of Health Economics (IHE), which is currently recognized as one of the tools to assess the evidence quality of case series studies. Additional file 2: Appendix 2 includes 20 entries in eight categories, including study objective, design, population, intervention and co-intervention, outcome measures, statistical analysis, results and conclusions, competing interests, and sources of support [40]. The assessment results are expressed “yes”, “no”, and “unclear”, representing low, high, and uncertain risk, respectively. For SRs/MAs, the evidence quality was evaluated using the AMSTAR 2 quality assessment tool, consisting of a total of 16 items in eight areas, including subject design, information retrieval, data extraction, data analysis, risk of bias assessment, description of results, comprehensive discussion, and conflict of interest. The results were expressed as “yes” and “no” [41].

### Statistical analysis

The ARs of stem cell therapy for KOA were analyzed using STATA/SE 16 software and expressed as effect sizes (ES) and their 95% CI. Using statistical *χ*^2^ test and *I*^2^ tests, heterogeneity was estimated and assumed among the included studies if (*P* < 0.1) and (*I*^2^ > 50%). Random-effects model was used for combined analysis. Otherwise, the fixed-effects model was used. Where there was duplication of included papers in different SRs/MAs or differences in literature sources, retrieval strategies, inclusion and exclusion criteria, and data extraction and analysis methods, combining them for analysis may be misleading. Therefore, we only performed a descriptive analysis (results are presented in a forest plot) and avoided pooled analysis of SRs/MAs results.

## Results

### Literature search results

**Fig. 1:**
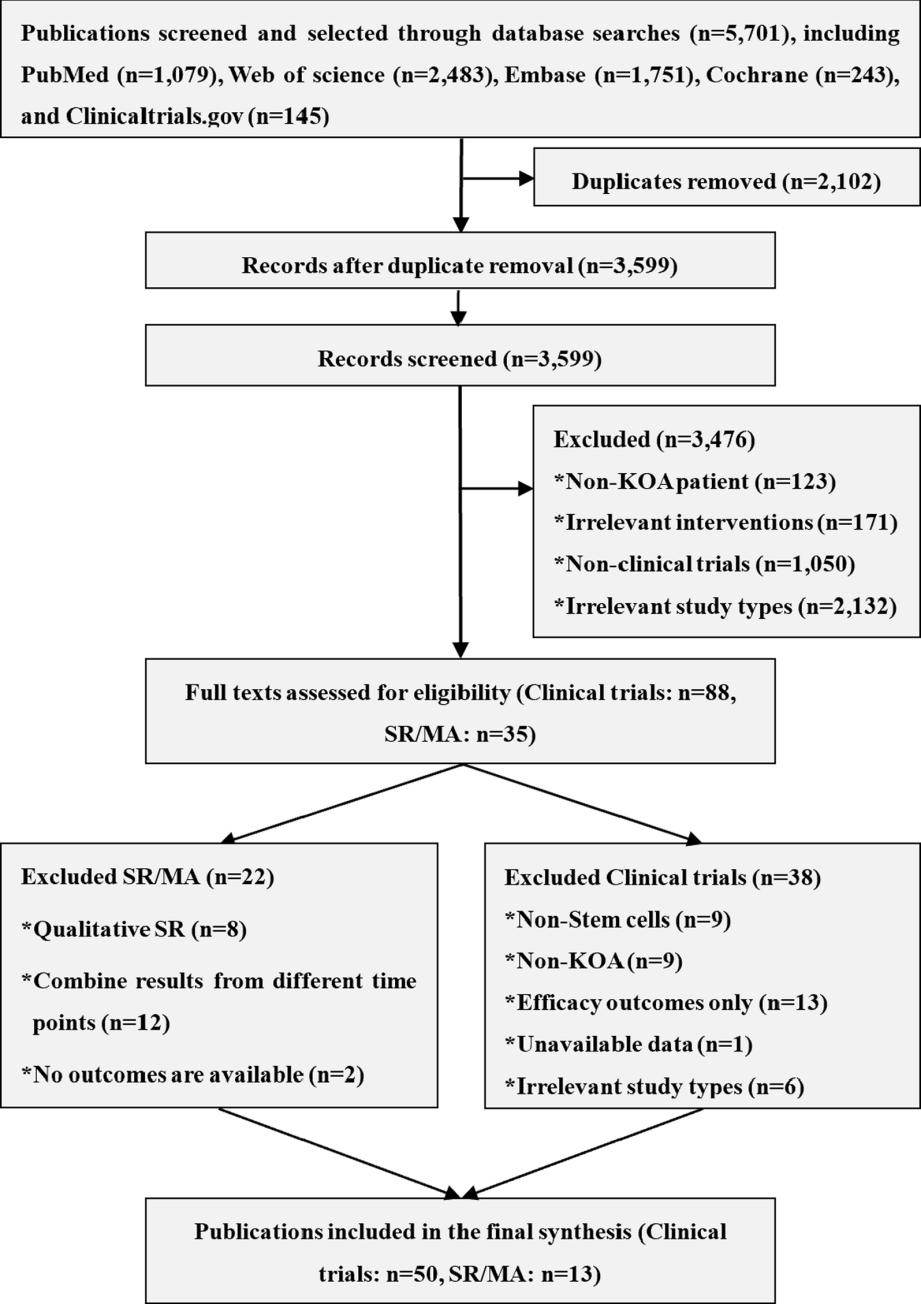
PRISMA flow chart

Following the initial and updated searches, 5701 pieces of literature were obtained. We excluded 123 non-KOA studies, including cartilage injury, anterior cruciate ligament injury, and hip osteoarthritis; 1050 non-clinical studies, including animal studies and cell experiments; 171 non-stem cell therapy studies involving platelets, plasma, bone marrow concentrate, adipocyte tissue, and vascular matrix components; and 2132 studies of irrelevant research studies including reviews, case reports, letters, conference abstracts. A total of 50 clinical trials reporting ARs of stem cell therapy on KOA and 13 SRs/MAs of stem cell therapy on KOA were included (Fig. 1).

### Basic details about the included studies

Included in the 50 studies were 16 RCTs [42–57], 10 controlled trials [58–67], and 24 single-arm studies [68–91]. There were 1223 patients, including 565 men and 807 women (sexes ratio was missed in four studies). A single study included 8 [72]—329 [85] patients, whose ages ranged from 20 [85]—80 [56] years and whose K–L scores ranged from 1 to 4. Types of stem cells studies were Adipose tissue-derived mesenchymal stem/stromal cells (ADMSCs; 23 studies), bone marrow-derived mesenchymal stem/stromal cells (BMSCs; 15 studies), umbilical cord mesenchymal stem/stromal cells (UCMSCs; 10 studies), placental mesenchymal stem/stromal cells (PLMSC; one study), and synovial tissue MSCs (one study). The stem cells sources included autologous adipose tissue (22 studies), autologous bone marrow tissue (14 studies), autologous synovial tissue (one study), allogeneic adipose tissue (one study), allogeneic bone marrow tissue (one study), allogeneic human placenta (one study) and allogeneic human umbilical cord (10 studies). The transplantation dose of stem cells ranged from 3 × 10^5^ [79] to 1 × 10^8^ [54], while the transplantation dose of stem cells was missing in nine studies. The frequency of stem cell administration was once (47 studies) and twice (three studies). The methods of stem cell administration included direct injection into the articular cavity (43 studies), hyaluronic acid hydrogel (six studies), and fibrin scaffold (one study). The follow-up period varied across studies, ranging from 6 months [53] to 120 months [60] (Additional file 1: Table S2 of Appendix 1).

The studies in 11 of the 13 SRs/MAs were RCTs [92–102], while two included RCTs and Non-RCTs [103, 104]. The analysis included patients ranging between 138 [100] and 1494 [102]. MSCs included in this analysis were ADMSCs, BMSCs, and UCMSCs. Only eight studies reported stem cell transplantation doses [93, 98–104], ranging from 1 × 10^6^ to 1.5 × 10^8^. Three to 10 electronic databases were searched. One study did not report PRISMA guidelines [95]. All studies were evaluated for quality. All nine studies that reported ARs after stem cell transplantation [92, 94, 96, 97, 99, 101–104] treated ARs as a secondary indicator without calculating the ARs incidence and explored the impact of ARs on patients (Additional file 1: Table S3 of Appendix 1).

### Meta-analysis results

#### Safety outcomes (based on clinical trials)

Nineteen ARs were reported in 50 studies, and MAs were performed. The highest incidence of increased knee pain among the five knee-related ARs was 24.5%. The incidence of the other six common ARs was less than 5%, except for muscle pain, which was 7.9%. Additionally, seven ARs were reported in only one study, among which prostate cancer and breast tumor were insignificant ARs, although they were observed during the follow-up period (Table 1).

**Table 1:** Incidence of adverse reactions

ARs	Number of studies	Incidence rate and its 95%CI	Heterogeneity (*I*^2^)
*Knee symptoms*			
Increased knee pain	36	24.5% [14.7%, 35.7%]	92.54%
Increased knee joint effusion	22	13.0% [5%, 23.0%]	87.23%
Increased knee swelling	29	11.9% [3.5%, 23.5%]	95.27%
Increased knee stiffness	14	5.6% [0%, 19.5%]	95.49
Increased knee synovitis	10	2.5% [0%, 8.5%]	67.68%
*General symptoms*			
Muscle pain	14	7.9% [0.8%, 19.2%]	89.35%
Low Back Pain	12	4.8% [0.2%, 12.8%]	71.57%
Increased susceptibility to infection	14	3.8% [0.3%, 9.5%]	64.78%
Dizziness and irritability	12	0.6% [0%, 2.3%]	29.42%
Nausea	9	0.1% [0%, 1.5%]	2.69%
Allergic reactions	13	0% [0%, 0.7%]	10.58%
Abdominal discomfort	8	0% [0%, 1.1%]	0%
*Other symptoms*	Only one study	Events	Total people
Subchondral cyst		16	37
Prostate cancer		1	329
Breast tumor		1	12
Superficial phlebitis		1	16
Urinary tract infection		1	18
Urolithiasis		1	18
Urination disorder		1	7

#### Efficacy outcomes (based on SRs/MAs)

**Fig. 2:**
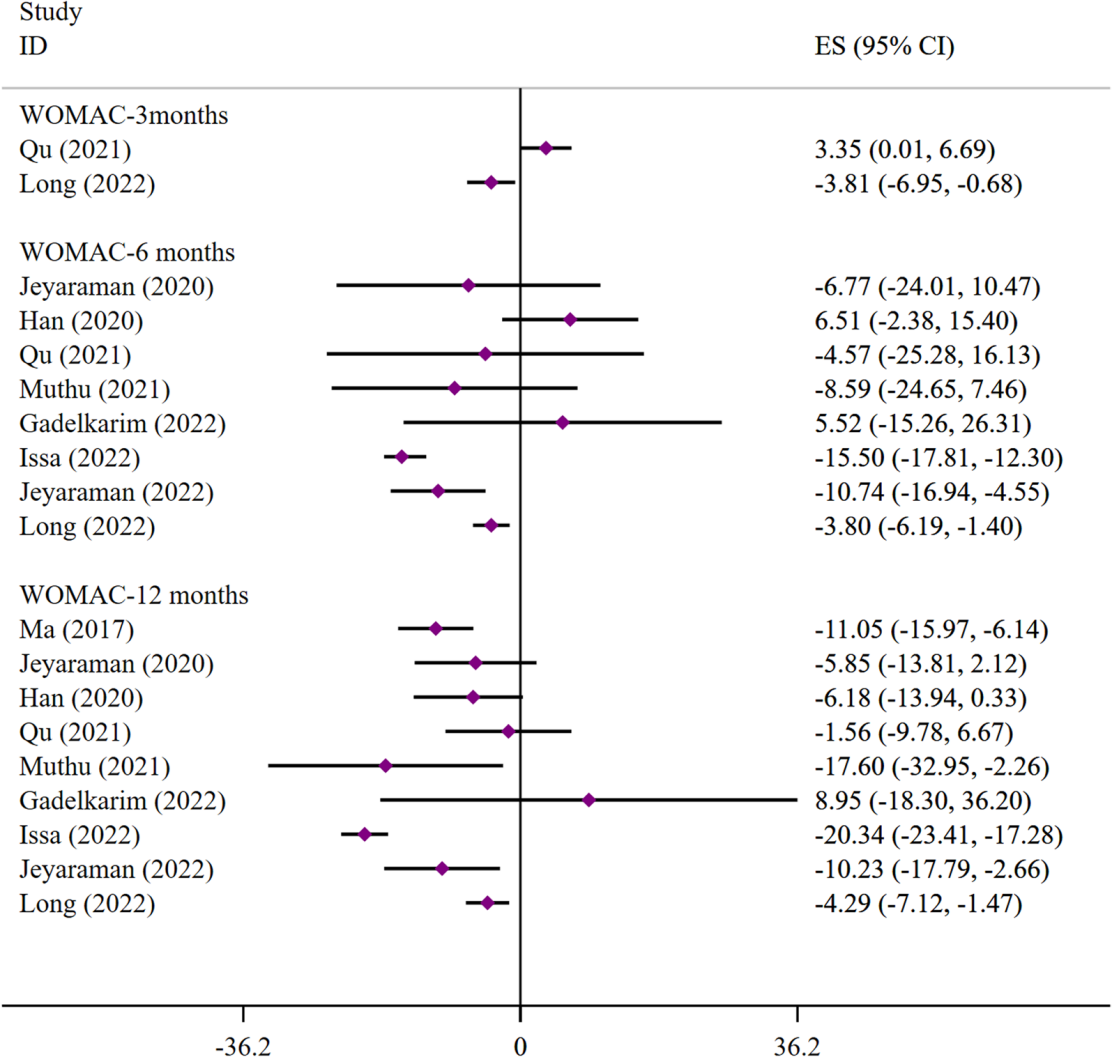
Meta-analysis results of WOMAC

**Fig. 3:**
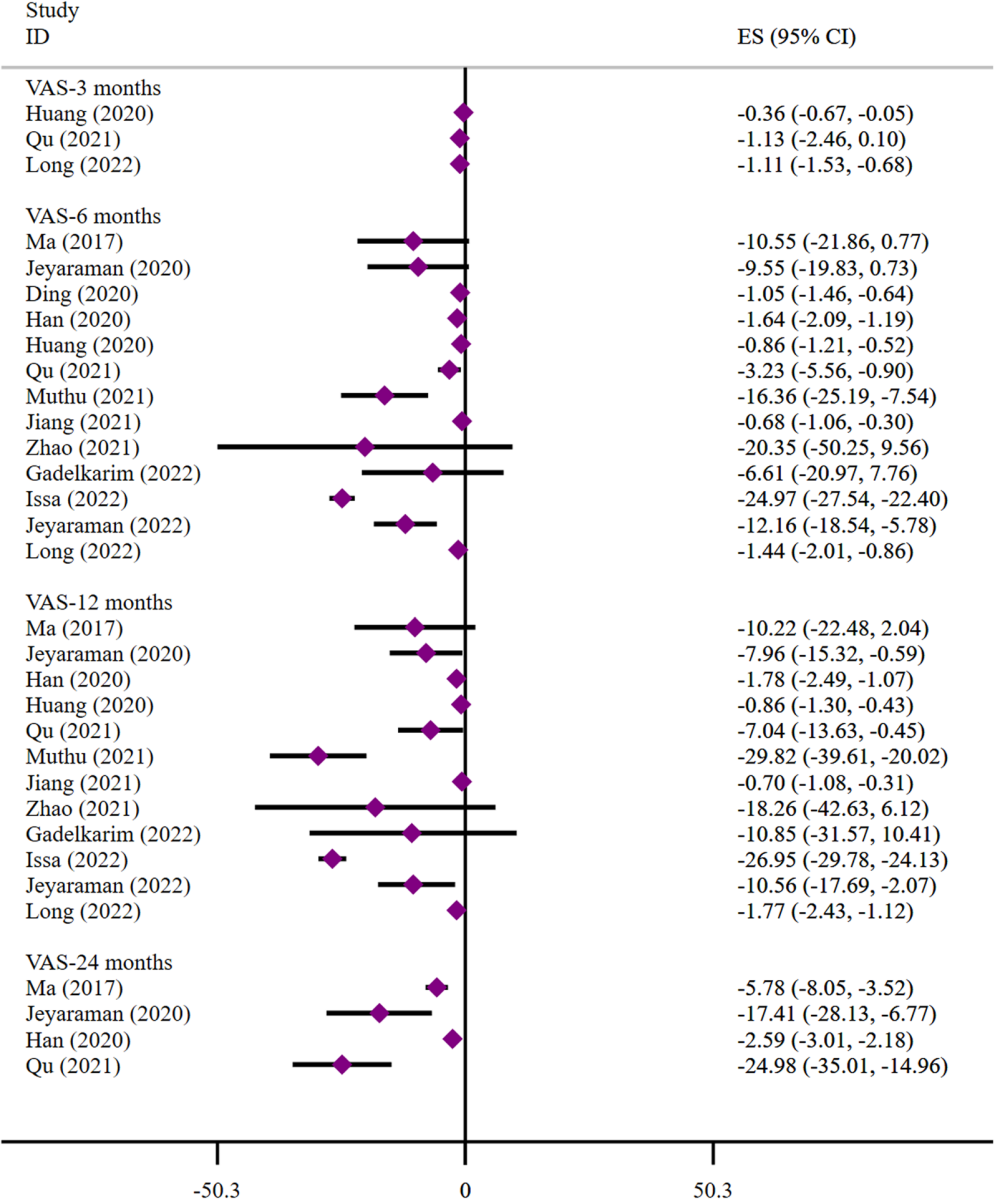
Meta-analysis results of VAS (At 24 months, all studies showed that stem cell therapy significantly improved VAS scores in KOA patients)

(1) WOMAC: Nine SRs/MAs reported WOMAC scores. At 3 months, the results from Qu et al. [93] showed that stem cells were less effective than conventional treatment in improving the patients WOMAC scores (WMD = 3.35; 95% CI [0.01, 6.69]). Conversely, Long et al. [105] showed that stem cells improved patients WOMAC scores better than conventional treatment (WMD = − 3.81; 95% CI [− 6.95, − 0.68]. At 6 months, only three studies [100, 101, 105] and at 12 months, only five [94, 100, 101, 103, 105] demonstrated that stem cells significantly improved patients WOMAC scores (Fig. 2).

(2) VAS: Thirteen SRs/MAs reported VAS scores. At 3 months, Huang et al. [97] and Long et al. [105] found that stem cell transplantation significantly improved patients VAS scores compared to the opposite results obtained by Qu et al. [93]. At 6 months, four studies concluded that stem cells did not improve the VAS scores of patients [92, 103, 104, 106]. At 12 months, three studies concluded that stem cells did not improve patients VAS scores [92, 103, 104]. At 24 months, all studies showed that stem cell transplantation significantly improved the patients VAS scores (Fig. 3).

### Quality assessment results

#### Clinical trials

**Fig. 4:**
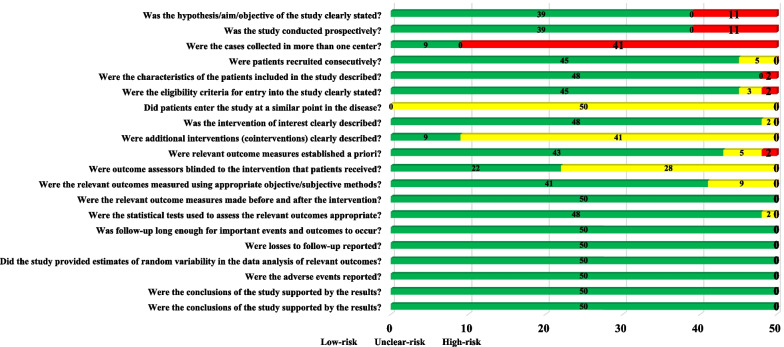
Quality assessment results of clinical trials

According to the IHE, 50 included studies showed a low risk of bias in 14 items, mainly in six areas of intervention and co-intervention, outcome measures, statistical analysis, results and conclusions, competing interests and sources of support. However, the studies had poor quality evidence for six items related to the study objective, design, and population. Reasons include 11 studies with unclear purpose and hypothesis, 11 with no prospective data collection, 41 with no report on concurrent treatments, 28 with no report on blinding of outcome evaluators, all with no report on similar severity of KOA in patients, and only nine studies conducted multicenter trials. (Fig. 4).

#### SRS/MAs

**Fig. 5:**
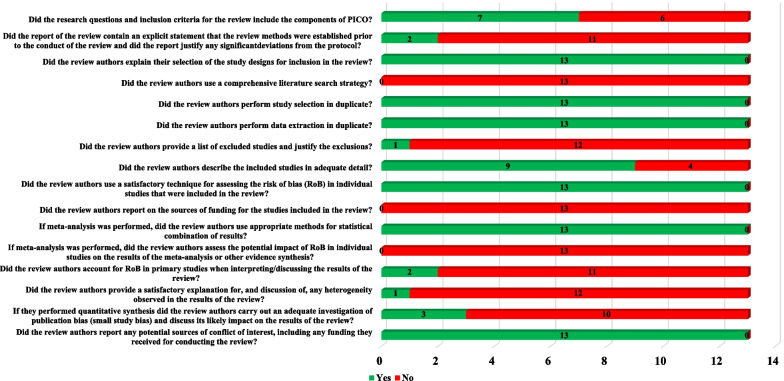
Quality assessment results of SRs/MAs

AMSTAR 2 [41] analysis of 13 studies revealed a low risk of bias in only six entries related to data search, extraction, and analysis. However, these studies lacked adequate evidence quality for nine items in five areas, including study design, bias risk assessment, outcome description, comprehensive discussion, and conflicts of interest. Reasons for these shortcomings included unclear reporting of PICOS principles in six studies, lack of protocol registration in 11 studies, failure to provide a list of excluded papers in 12 studies, insufficient result description in four studies, no reporting of funding sources in all 13 SRs/MAs, discussion of bias risk of included studies in only two SRs/MAs, and adequate description of publication bias effect on study results in only three SRs/MAs (Fig. 5).

#### Registration status of clinical studies on stem cell therapy for KOA

Out of 50 clinical studies that met inclusion criteria, only half prospectively registered with the clinical trial registration platform, indicating pessimistic current clinical trial registration status. To understand the current status of stem cell therapy in treating KOA, we analyzed 145 studies from Clinicaltrials.gov. Results showed 53 completed, but only four reported results (met the inclusion criteria and were included in the analysis). The possible reason is that the trial registrant may not have followed up on the registered protocols, or more potential papers have not been published. Further, 30 studies are recruiting patients, six studies are inactive (not recruiting), one study is no longer available, nine studies are still not recruiting, three studies have been terminated, 36 studies have unknown status, and seven studies have withdrawn.

Additionally, 85 of 145 clinical trials were RCTs. Among them, 42 studies were blind to participants and outcomes assessors, 13 studies were blind to participants only, nine studies were blind to outcomes assessors, and 21 studies were open-label. Conclusively, while numerous studies investigate stem cells safety and efficacy in treating KOA, only 28.97% of the clinical trials are randomized, double-blind RCTs, which is the gold standard for intervention quality assessment [107]. Thus, there is still a significant need for improvement in clinical trial design and result reporting for stem cell therapy in KOA treatment (Additional file 3: Appendix 3).

## Discussion

### Evidence overview of safety

The safety of stem cell therapies is critical in determining their potential introduction into clinical practice, even for non-life-threatening diseases. However, previous studies have not adequately focused on the ARs associated with stem cell transplantation. Some studies have reported ARs without calculating their incidence or severity, let alone analyzing their impact on patients in detail [108]. Knee-related symptoms, including pain, swelling, effusion, and stiffness, are the most commonly observed and reported ARs during stem cell transplantation [109]. However, it is generally accepted that there is a low probability of establishing a cause-effect relationship between these symptoms and stem cell transplantation. A multicenter analysis of 2372 patients with various orthopedic conditions has confirmed the safety of stem cell therapy, with an incidence of ARs such as pain and knee joint swelling at 12.1%, and severe ARs, such as tumors, neurological and vascular events, at 1.5%, with tumors accounting for only 0.3% (related to the patient's condition) [110]. Our study revealed a high incidence of knee-related symptoms, with increased knee pain after stem cell transplantation as high as 24.5%.

Furthermore, there is a growing body of evidence indicating that synovial inflammation plays a significant role in the symptomatic and structural progression of knee osteoarthritis (KOA). Synovitis has been linked to symptom severity, cartilage degeneration, and bone redundancy formation [111]. However, our study found a low incidence of synovitis after stem cell injection, at only 2.5%. Although no serious ARs have been observed, it is critical to take these common ARs seriously and minimize their occurrence by continually optimizing stem cell collection and transplantation procedures. Among the 50 clinical studies analyzed, 38 obtained stem cells from autologous bone marrow or adipose tissue. Although both procedures are relatively simple with minimal side effects, 7.9% of patients experienced pain at the collection site following bone marrow collection. A national survey of 112,756 patients revealed that only 0.1% of liposuction patients experienced complications [112]. This aligns with our result that there is virtually no abdominal discomfort following abdominal liposuction (ES = 0%; 95% CI [0%, 1.1%]).

Additionally, 47 of 50 studies analyzed had a short-term follow-up period of 1–2 years, which may be insufficient to identify complications related to cell differentiation. Therefore, the long-term effects of stem cell therapy remain uncertain. Notably, two among 50 studies reported cancer diagnoses in patients during follow-up periods after stem cell therapy: Freitag et al. reported one case of prostate cancer during a 2-year follow-up period [85], while Chen et al. reported one case of breast tumor during a 48-week follow-up period [88]. However, both studies concluded that stem cell therapy did not have significant ARs on patients. While they all agreed that tumorigenesis and stem cell therapy have no direct link, they did not provide supporting evidence. While it may be implausible that stem cells directly contribute to tumorigenesis, there is evidence that they can regulate tumorigenesis and progression and even be a source of tumors [22]. In a mouse melanoma model, it was found that MSCs can migrate to tumor sites and support the proliferation, invasion, and metastasis of tumor cells [113]. Various studies have indicated that human MSCs may facilitate the proliferation of tumor cells [114–116]. Although this effect is generally irrelevant to KOA, we must consider that KOA and neoplastic diseases have similarities, like a strong correlation with age. Therefore, a carcinogenic or pro-carcinogenic role of stem cells in KOA is possible.

Moreover, some studies have reported chromosomal abnormalities in MSCs, which may undergo a malignant transformation in vitro [117, 118]. Therefore, it is necessary to clarify how stem cells promote tumorigenesis and progression in cancer and KOA patients. Finally, tumor formation can take years, but some studies overconfidently claimed the safety of stem cell therapy after only 2–3 years of follow-up and should enter the clinic immediately to benefit patients, which is biased [119, 120]. Although no complications have been reported, longer follow-up studies involving a wider range of patients are required to ensure the safety of these procedures.

### Evidence overview of efficacy

High-quality studies supported by SRs/MAs are essential to inform clinical decisions. Our systematic summary of published SRs/MAs found that extending follow-up time from 3 to 24 months resulted in gradual improvement in pain scores for most patients, but WOMAC scores consistently yielded conflicting results across different SRs/MAs [93, 105]. Possible reasons for this discrepancy include the limited sample sizes of the studies, the influence of individual studies on findings, and heterogeneity between different studies [94]. Additionally, existing SRs/MAs overlook that MSCs from different tissues or even the same tissue have distinct transcriptomic patterns and immunophenotypic characteristics, which may account for inexplicable and conflicting conclusions [121, 122]. Current research lacks standardization in stem cell acquisition approaches, cell dose and application, and consistency in control groups. For instance, some studies have used arthroscopic debridement, various doses of platelet-rich plasma, and collagen gel as controls [81, 95]. Additionally, certain studies lacked baseline data like age range and K–L classification of patients, which may introduce bias in evaluating the efficacy of stem cell therapy [95]. Notably, stem cell therapy has shown efficacy in preventing or limiting the progression of KOA in the early stages, with insignificant efficacy in the later stages [81]. However, given that current clinical studies encompass patients with varying degrees of disease severity, conflicting results may arise.

The published SRs/MAs may not support clinical decisions either. First, there are differences in the included papers between SRs/MAs due to different inclusion/exclusion criteria, search strategies, and others. Second, stem cell type, transplantation dose, injection frequency, KOA severity, and follow-up time vary widely among clinical studies, affecting results. However, data analysis by SRs/MAs ignored these factors [98]. The varying outcomes across different SRs/MAs precisely reflect the differences in outcomes of the included clinical studies, suggesting that the role of stem cell therapy in KOA treatment is yet unclear. These controversial findings do not support health and social care decisions, and definitive conclusions on stem cell use for KOA cannot be made with absolute certainty.

### Evidence overview of study quality

This situation is made worse by the poor quality of evidence from clinical trials and SRs/MAs, as neither the efficacy nor safety of stem cells is promising. Most of the 50 included studies were case reports or case series studies. These studies provided little valuable information. Further, the absence of a control group made it impossible to determine whether the patients improvement resulted from the transplanted stem cells or another source. Due to the limitations of such studies, patients are rarely followed for longer than 2 years [74, 82, 83]. Neither potential benefits nor risks are observed. The results of included SRs/MAs showed that stem cells improved the pain scores of patients only 24 months after transplantation, and there was an insignificant difference between the therapeutic effect of stem cells and placebo in the 2 years prior. Only four of the 13 SRs/MAs studies investigated 2-year follow-up outcomes.

Moreover, 28 studies were unblinded to outcome evaluators and fewer blinded patients. Patients and evaluators have high expectations for stem cell therapies, which may result in significant bias in data collection and reporting. Although 96% of the studies described the type, source, and transplantation dose of stem cells, many patients received co-treatments such as arthroscopic debridement, microfracture, and high tibial osteotomy. These confounding factors can prevent an equitable comparison of stem cell efficacy. However, 84% of the studies did not report whether patients received other treatments besides stem cells, preventing a clear understanding of the actual contribution and clinical potential of stem cell-based products. In 43 studies, knee severity ranged from grades 1–4 for patients. Stem cell therapy may be more effective in the early stages of KOA because inflammation levels are lower than in the late stages [123]. It is more difficult to determine stem cell efficacy based on a comprehensive analysis of patients with varying severity.

The Osteoarthritis Research Society International (OARSI) does not recommend stem cell therapy for treating KOA due to the low evidence quality of clinical studies [12], and guidelines from other countries agree that the stem cells efficacy should be confirmed by higher quality studies [124, 125] showing consistency with our findings. Despite creating PRISMA guidelines to standardize the production and reporting of SRs/MAs, 12 out of 13 studies claimed adherence to these guidelines. However, our analysis indicates that following PRISMA guidelines may not necessarily enhance the methodological quality of the study. This may be because PRISMA reporting specification merely provides researchers with a checklist of items to report while they themselves determine the actual content and level of detail of the final report. Additionally, the list of excluded studies and their reasons, and the early registration of protocols, had a substantial effect on the methodological quality of SRs/MAs [126], but few studies followed guidelines by registering protocols, establishing PICOs, and providing lists of excluded papers. According to AMSTAR 2 and PRISMA guidelines, relying solely on electronic database searches is insufficient. It should be supplemented by searches of clinical trial registries, gray literature (literature that has not been officially published and distributed, belonging to non-mainstream literature, mostly including non-profit government publications, dissertations, conference literature, scientific reports, teaching courseware, preprints [127].), consulting with field experts, and other measures, as studies with negative results, are often difficult to publish [128]. None of the 13 included studies in our analysis searched beyond electronic databases, raising concerns about data comprehensiveness. Quality of evidence affects the accuracy and reliability of SRs/MAs [129]. Despite all 13 SRs/MAs assessing the quality of clinical studies included, none evaluated its impact on findings. Thus, low-quality clinical studies and SRs/MAs offer limited assistance in translating stem cell research into clinical practice.

### Limitations

(1) Insufficient reporting of additional medications or surgical procedures for patients who received stem cell therapy may have affected the analysis results. (2) The absence of control groups in the single-arm meta-analysis on stem cell safety makes it unclear whether adverse reactions were due to the stem cells or the transplantation procedures. (3) Unclear reporting of the stem cell transplantation dose in the included study prevented a subgroup analysis based on the transplantation dose of stem cells. (4) Most studies reported a relatively short follow-up period, hindering the assessment of the long-term effects of stem cells. (5) Majority of studies were small (had < 20 patients), which, given the high variability among patients, hinders the ability to assess rare but significant side effects and draw meaningful conclusions from such a small study.

### Current challenges and future perspectives

Although the clinical application of stem cell therapy for KOA is promising, there are still too many problems to be resolved. (1) There is no standard procedure for isolating, cultivating, and expanding stem cells. A reproducible, standard procedure must be established to bring hope to the KOA treatment. (2) The type and source, transplantation dose, and a number of stem cell injections impact the therapeutic efficacy of KOA [130]. To bridge the gap between patient expectations and clinical applications of stem cell therapies, standardized protocols must be developed to determine the optimal stem cell transplantation strategy. (3) The included clinical trials did not address whether autologous stem cells undergo transdifferentiation after in vitro culture, whether allotransplanted stem cells produce excessive transplant rejection, or whether gender mismatch affects the outcomes. (4) In the current study, the K–L of patients ranged from grades 1–4 and was not stratified by important risk factors, including age or BMI, making it difficult to quantify the stem cells effectiveness accurately [131]. Furthermore, at what KOA degeneration stage stem cell therapy is optimally administered is unclear. (5) Current criteria for the diagnosis and efficacy evaluation of KOA are not uniform, and scoring systems such as WOMAC and VAS are non-specific and significantly influenced by patient and investigator preferences. To facilitate a more accurate comparison of studies to determine the most effective treatment for KOA, optimizing and harmonizing the assessment criteria using a small number of scoring systems instead of multiple systems [132]. (6) MSCs and cell concentrates are different products, but their nomenclature is frequently confused in the scientific literature. Distinguishing between commonly used cell concentrates and laboratory-purified stem cells is necessary to clarify the efficacy of actual stem cells [133, 134]. (7) Most current clinical studies are case reports, case series, non-randomized controlled studies, or unblinded randomized controlled trials. These poor-quality studies provide scant evidence to confirm the efficacy and safety of stem cell therapy. Therefore, future clinical trials should be placebo-controlled, randomized, and blind (participants, outcome assessors). Additionally, KOA patients with the same severity should be recruited from as many centers as possible. (8) Currently, stem cell therapy for KOA is undergoing a crucial clinical transition, and many preclinical studies are associated with it [20]. Like clinical trials, preclinical data from randomized, blind, and large samples are undoubtedly reliable. Following a standardized experimental design, preclinical studies should carefully choose animal models that most resemble human KOA. It is essential to investigate the internal mechanisms of stem cell function, determine the efficacy of different stem cell sources, and identify the optimal dosage, mode, and timing of stem cell administration to enhance therapeutic outcomes to advance the clinical transformation of stem cell therapy in the future.

## Conclusions

As stem cell therapies are on the cusp of clinical implementation, a thorough examination and deliberation of the safety, efficacy, and study evidence quality regarding the clinical translation of stem cells are crucial to enhance benefits and minimize risks for KOA patients. Our analysis of 50 clinical studies and 13 SRs/MAs revealed that inconsistent effectiveness outcomes, potential safety risks, and poor evidence quality hinder any recommendation for stem cell product use in KOA patients. Maintaining a critical view of innovative stem cell therapies and establishing standards in legislation, clinical trial management, and processing is imperative to ensure comparability between trials. Therefore, we conclude that the clinical translation of stem cell therapies for KOA lacks sufficient support and should be approached cautiously until stronger evidence is available.

## Supplementary Information

**Additional file 1: Appendix 1.** **Appendix 1. Table S1.** Search strategies. **Table S2.** Basic information of the included clinical studies. **Table S3.** Basic information of the included SRs/MAs.

**Additional file 2: Appendix 2.** Quality appraisal checklist for case series studies and instructions for use.

**Additional file 3: Appendix 3.** Registration status of clinical studies on stem cell therapy for KOA.

## Data Availability

The datasets used and/or analyzed during the current study are available from the corresponding author on reasonable request.

## Abbreviations

KOA: Knee osteoarthritis
IHE: Institute of Health Economics
SRs/MAs: Systematic reviews/meta-analyses
NSAIDs: Non-steroidal anti-inflammatory drugs
ARs: Adverse reactions
RCTs: Randomized controlled trials
MSCs: Mesenchymal stem/stromal cells
PRISMA: Preferred reporting items for systematic reviews and meta-analyses
KOOS: Knee osteoarthritis outcome score
WOMAC: Western Ontario and McMaster Universities Osteoarthritis Index
IKCD: International Knee Documentation Committee
VAS: Visual analog scale
K–L: Kellgren–Lawrence
ES: Effect sizes
ADMSCs: Adipose tissue-derived mesenchymal stem/stromal cells
BMSCs: Bone marrow-derived mesenchymal stem/stromal cells
UCMSCs: Umbilical cord mesenchymal stem/stromal cells
PLMSCs: Placental mesenchymal stem/stromal cells
