# Supplementary material for: A systematic review, umbrella review, and quality assessment on clinical translation of stem cell therapy for knee osteoarthritis: Are we there yet?
Source: Stem Cell Res Ther. 2023 Apr 15;14:91. doi: 10.1186/s13287-023-03332-5 (PMC10105961; doi:10.1186/s13287-023-03332-5)
Supplement: Supplementary file 1 — Additional file 1: Appendix 1. Appendix 1. Table S1. Search strategies. Table S2. Basic information of the included clinical studies. Table S3. Basic information of the included SRs/MAs. [file 13287_2023_3332_MOESM1_ESM.docx]

**A Systematic Review, Umbrella Review, and Quality Assessment on Clinical Translation of Stem Cell Therapy for Knee Osteoarthritis: Are We There Yet?**

**Table 1: Search strategies**

| **1. PubMed**  #1: "Stem Cells"[MeSH Terms] 251,136  #2: "stem cell"[Title/Abstract] OR "stem cells"[Title/Abstract] 318,970  #3: #1 OR #2 411,585  #4: "knee osteoarthritis"[Title/Abstract] OR ("knee"[Title/Abstract] AND "osteoarthritis"[Title/Abstract]) 33,823  #5: "osteoarthritis, knee"[MeSH Terms] 25,304  #6: #4 OR #5 41,290  #7: #3 AND #6: 889  **2. WOS**  (TS=('stem cell' OR 'stem cells')) AND TS=('knee osteoarthritis' OR (knee AND osteoarthritis)) 2343  **3. Cochrane**  #1: (stem cell OR stem cells):ti,ab,kw 14633  #2: MeSH descriptor: [Stem Cells] explode all trees 934  #3: #1 or #2 14739  #4: (knee osteoarthritis OR (knee AND osteoarthritis)):ti,ab,kw 14423  #5: MeSH descriptor: [Osteoarthritis, Knee] explode all trees 5183  #6: #4 or #5 14423  #7: #3 and #6 233  **4.** **Ovid Embase**  #1: 'stem cell':ab,ti OR 'stem cells':ab,ti 459126  #2: 'stem cell'/exp 441249  #3: #1 OR #2 602911  #4: 'knee osteoarthritis':ab,ti OR (knee:ab,ti AND osteoarthritis:ab,ti) 50242  #5: 'knee osteoarthritis'/exp 41503  #6: 'knee'/exp 78031  #7: 'osteoarthritis'/exp 153791  #8: #6 AND #7 15286  #9: #4 OR #5 OR #8 64313  #10: #3 AND #9 1530 |
| --- |

**Table 2: Basic information of the included clinical studies**

| **Author** | **Year** | **Country** | **Study Type** | **Sample size** | **Age** | **Gender (M/F)** | **K-L level** | **Stem cells** | | | | | **Follow-up time** |
| --- | --- | --- | --- | --- | --- | --- | --- | --- | --- | --- | --- | --- | --- |
|  |  |  |  |  |  |  |  | Source | Sort | Dose | Method of administration | Frequency |  |
| Soltani | 2019 | Iran | RCT | 10 | 57.5 | 1/9 | 2-4 level | Allogeneic placenta | PLMSC | 5-6×10^7^ | Intra-articular injection | 1 | 24 weeks |
| Chen | 2022 | China | Single arm study | 12 | 50-75 | 3/9 | 2-3 level | Autologous adipose tissue | ADMSCs | 5×10^7^ | Intra-articular injection | 1 | 48 weeks |
| Yokota | 2019 | Japan | Cohort study | 42 | 70±9.1 | 9/33 | 2-4 level | Autologous adipose tissue | ADMSCs | 1.28×10^7^ | Intra-articular injection | 1 | 6 months |
| Chen | 2021 | China | RCT | 57 | 40-80 | 3/12 | 1-3 level | Autologous adipose tissue | ADMSCs | 6.4×10^7^ | Intra-articular injection | 1 | 96 weeks |
| Kim | 2020 | Korea | Control | 30 | 57-70 | 11/19 | / | Autologous adipose tissue | ADMSCs | 6×10^6^ | Intra-articular injection | 1 | 1 year |
| Sekiya | 2021 | Japan | Single arm study | 8 | ≥20 | 3/5 | 2-4 level | Autologous synovial tissue | Synovial MSCs | 2×10^7^ | Intra-articular injection | 2 | 30 weeks |
| Kim | 2014 | Korea | Control | 37 | 57.5±5.9 | 14/23 | 1-2 level | Autologous adipose tissue | ADMSCs | 3.9×10^6^ | Fibrin scaffold | 1 | 29.2±4.1 months |
| Jo | 2017 | Korea | Cohort study | 18 | 52-72 | 3/15 | 3-4 level | Autologous adipose tissue | ADMSCs | 1-10×10^7^ | Intra-articular injection | 1 | 2 years |
| Orozco | 2013 | Spain | Single arm study | 12 | 49±5 | 6/6 | 2-3 level | Autologous bone marrow tissue | BMSCs | 4×10^7^ | Intra-articular injection | 1 | 1 year |
| Koh | 2012 | Korea | Single arm study | 18 | 41-69 | 6/12 | 3-4 level | Autologous adipose tissue | ADMSCs | 0.3-2.7×10^6^ | Intra-articular injection | 1 | 2 years |
| Emadedin | 2018 | Iran | RCT | 19 | 51.7±9.2 | 12/7 | 2-4 level | Autologous bone marrow tissue | BMSCs | 4×10^7^ | Intra-articular injection | 1 | 6 months |
| Koh | 2012 | Korea | Case-control study | 25 | 34-69 | 8/17 | 1-3 level | Autologous adipose tissue | ADMSCs | 1.89×10^6^ | Intra-articular injection | 1 | 16.4 months |
| Soler | 2015 | Spain | Single arm study | 15 | 33-63 | 6/9 | 2-3 level | Autologous bone marrow tissue | BMSCs | 4.09×10^7^ | Intra-articular injection | 1 | 1 year |
| Song | 2020 | Korea | Case series study | 128 | 40-78 | 42/86 | 1-3 level | Allogeneic human umbilical cord | hUCB-MSCs | 7.5×10^6^ | Intra-articular injection | 1 | 2 years |
| Higuchi | 2020 | Japan | Single arm study | 34 | / | / | 3-4 level | Autologous adipose tissue | ADMSCs | 1×10^8^ | Intra-articular injection | 1 | 6 months |
| Spasovski | 2018 | Serbia | Single arm study | 9 | 39-78 | 3/6 | 2-4 level | Autologous adipose tissue | ADMSCs | 0.5-1×10^7^ | Intra-articular injection | 1 | 18 months |
| Song | 2018 | China | Control | 36 | 40-70 | 4/14 | 2-4 level | Autologous adipose tissue | ADMSCs | 1-5×10^7^ | Intra-articular injection | 1 | 96 weeks |
| Freitag | 2018 | Australia | RCT | 20 | 54.6±6.3 | / | 2-3 level | Autologous adipose tissue | ADMSCs | 1×10^8^ | Intra-articular injection | 1 | 1 year |
| Lu | 2020 | China | RCT | 22 | 59.29±4.14 | 3/19 | 2-3 level | Allogeneic adipose tissue | ADMSCs | 1-5×10^7^ | Intra-articular injection | 2 | 48 weeks |
| Samara | 2021 | Jordan | Single arm study | 16 | 42-73 | / | 3-4 level | Allogeneic human umbilical cord | hUCB-MSCs | 8.62×10^7^ | Intra-articular injection | 1 | 4 years |
| Freitag | 2022 | Australia | Case series study | 329 | 58.6±11.8 | 199/130 | 1-4 level | Autologous adipose tissue | ADMSCs | 5×10^7^ | Intra-articular injection | 1 | 2 years |
| Koh | 2013 | Korea | Single arm study | 30 | 65-80 | 5/25 | 1-3 level | Autologous adipose tissue | ADMSCs | 4.04×10^6^ | Intra-articular injection | 1 | 2 years |
| Bastos | 2018 | Portugal | RCT | 18 | 57.6 ± 9.6 | 9/9 | 2-4 level | Autologous bone marrow tissue | BMSCs | 4×10^7^ | Intra-articular injection | 1 | 1 year |
| Yang | 2022 | Korea | Control | 55 | 56.4±5.3 | 13/42 | 3 level | Allogeneic human umbilical cord | hUCB-MSCs | / | Intra-articular injection | 1 | 33 months |
| Panni | 2018 | Italy | Single arm study | 52 | 37-78 | 22/30 | 1-2 level | Autologous adipose tissue | ADMSCs | / | Intra-articular injection | 1 | 15.3 months |
| Chung | 2020 | Korea | Case series study | 93 | 43-65 | / | 3 level | Allogeneic human umbilical cord | hUCB-MSCs | / | HA hydrogel | 1 | 1.7 years |
| Song | 2020 | Korea | Single arm study | 25 | 64.9 ± 4.4 | 2/23 | 2-4 level | Allogeneic human umbilical cord | hUCB-MSCs | / | Intra-articular injection | 1 | 2 years |
| Kuah | 2018 | Australia | RCT | 16 | 40-65 | 11/5 | 1-3 level | Autologous adipose tissue | ADMSCs | 3.9-6.7×10^6^ | Intra-articular injection | 1 | 1 year |
| Al-Najar | 2017 | Japan | Single arm study | 13 | 34-63 | 6/7 | 2-3 level | Autologous bone marrow tissue | BMSCs | 6.1×10^7^ | Intra-articular injection | 2 | 2 years |
| Lu | 2019 | China | RCT | 26 | 55.03±9.19 | 3/23 | 1-3 level | Autologous adipose tissue | ADMSCs | 5×10^7^ | Intra-articular injection | 1 | 1 year |
| Matas | 2018 | Chile | RCT | 18 | 56.1±6.8 | 7/11 | 1-3 level | Allogeneic human umbilical cord | hUCB-MSCs | 2-4×10^7^ | Intra-articular injection | 1 | 1 year |
| Lee | 2019 | Korea | RCT | 12 | 62.2±6.5 | 3/9 | 2-4 level | Autologous adipose tissue | ADMSCs | 1×10^8^ | Intra-articular injection | 1 | 6 months |
| Chahal | 2019 | Canada | Single arm study | 12 | 40-65 | 7/5 | 3-4 level | Autologous bone marrow tissue | BMSCs | 1-50×10^6^ | Intra-articular injection | 1 | 2 years |
| Pers | 2016 | France | Single arm study | 18 | 50-75 | 8/10 | 3-4 level | Autologous adipose tissue | ADMSCs | 2-50×10^6^ | Intra-articular injection | 1 | 6 months |
| Jo | 2013 | Korea | Single arm study | 18 | 63.3±8.6 | 3/15 | 3-4 level | Autologous adipose tissue | ADMSCs | 1-5×10^7^/1×10^8^ | Intra-articular injection | 1 | 6 months |
| Kim | 2022 | Korea | RCT | 13 | 58.3±6.4 | 2/11 | 2-4 level | Autologous adipose tissue | ADMSCs | 1×10^8^ | Intra-articular injection | 1 | 2 years |
| Kim | 2022 | Korea | Control | 11 | 52-74 | 3/8 | 2-4 level | Autologous adipose tissue | ADMSCs | 1×10^8^ | Intra-articular injection | 1 | 5 years |
| Vega | 2014 | Spain | RCT | 15 | 57±9 | 6/9 | 2-4 level | Allogeneic bone marrow tissue | BMSCs | 4×10^7^ | Intra-articular injection | 1 | 1 year |
| Garay-mendoza | 2017 | Mexican | Control | 30 | 55.67±12.02 | 7/23 | 2-3 level | Autologous bone marrow tissue | BMSCs | / | Intra-articular injection | 1 | 6 months |
| Lamo‑Espinosa | 2020 | Spain | RCT | 30 | 40-62 | 17/13 | 2-4 level | Autologous bone marrow tissue | BMSCs | 1×10^8^ | Intra-articular injection | 1 | 1 year |
| Lamo‑Espinosa | 2018 | Spain | RCT | 16 | 54.4-69.5 | 10/6 | 1-4 level | Autologous bone marrow tissue | BMSCs | 1×10^6^, 1×10^8^ | Intra-articular injection | 1 | 1 year |
| Park | 2016 | Korea | Single arm study | 7 | 29-77 | 2/5 | 3 level | Allogeneic human umbilical cord | hUCB-MSCs | 2.5×10^6^/cm2 | HA hydrogel | 1 | 24 weeks |
| Lamo‑Espinosa | 2016 | Spain | RCT | 20 | 50-80 | 12/8 | 2-4 level | Autologous bone marrow tissue | BMSCs | 1×10^6^, 1×10^8^ | Intra-articular injection | 1 | 1 year |
| Gobbi | 2019 | Italy | Case series study | 23 | 48.5±9.2 | 15/8 | 2-4 level | Autologous bone marrow tissue | BMSCs | / | HA hydrogel | 1 | 6 years |
| Emadedin | 2012 | Iran | Case series study | 6 | 54.56 | 0/6 | 4 level | Autologous bone marrow tissue | BMSCs | 2-2.4×10^7^ | Intra-articular injection | 1 | 1 years |
| Lim | 2021 | Korea | RCT | 43 | 55.3±8.9 | 15/28 | 4 level | Allogeneic human umbilical cord | hUCB-MSCs | 7.5×10^6^ | HA hydrogel | 1 | 1 years |
| Teo | 2019 | Singapore | Control | 36 | 43.5±11.2 | 20/16 | 3-4 level | Autologous bone marrow tissue | BMSCs | / | Intra-articular injection | 1 | 10 years |
| Ryu | 2020 | Korea | Control | 27 | 53.93±8.6 | 11/16 | 4 level | Allogeneic human umbilical cord | hUCB-MSCs | / | HA hydrogel | 1 | 2 years |
| Neckar | 2022 | Czech Republic | Case series study | 6 | 48±7 | 4/2 | 3-4 level | Autologous bone marrow tissue | BMSCs | / | HA hydrogel | 1 | 1 year |
| Günay | 2022 | Turkey | Case series study | 10 | 58.2±10.0 | 7/3 | / | Allogeneic human umbilical cord | hUCB-MSCs | 1×10^8^ | Intra-articular injection | 1 | 1 year |

**Table 3: Basic information of the included SRs/MAs**

| **Author** | **Year** | **Type of Study** | **Number of studies** | **Sample size** | **Stem cells** | **Dose** | **Databases searched** | **PRISMA** | **Quality assessment** |
| --- | --- | --- | --- | --- | --- | --- | --- | --- | --- |
| Gadelkarim | 2022 | Random/non-random | 15 | 463 | MSCs | 1-5×10^7^ | 6 | Yes | Cochrane Collaboration’s ROB2 tool/ROBINS-I |
| Jeyaraman | 2020 | Random | 19 | 811 | MSCs | / | 3 | Yes | Cochrane Collaboration’s ROB2 tool |
| Qu | 2021 | Random | 9 | 476 | MSCs | 5×10^6^-1.5×10^8^ | 6 | Yes | Cochrane Collaboration’s ROB2 tool |
| Ding | 2020 | Random | 13 | 377 | MSCs | 3.9×10^6^-1.5×10^8^ | 3 | Yes | Cochrane Collaboration’s ROB2 tool |
| Muthu | 2021 | Random | 17 | 767 | MSCs | / | 4 | Yes | Cochrane Collaboration’s ROB2 tool |
| Han | 2020 | Random | 9 | 377 | MSCs | 1.89-2×10^8^ | 5 | Yes | Cochrane Collaboration’s ROB2 tool |
| Ma | 2017 | Random/non-random | 11 | 521 | MSCs | 1×10^6^-1.5×10^8^ | 4 | Yes | Jadad scale |
| Jiang | 2021 | Random | 9 | 406 | MSCs | / | 7 | No | Cochrane Collaboration’s ROB2 tool |
| Huang | 2020 | Random | 9 | 339 | MSCs | / | 3 | Yes | Cochrane Collaboration’s ROB2 tool |
| Zhao | 2021 | Random | 6 | 251 | MSCs | / | 6 | Yes | Cochrane Collaboration’s ROB2 tool |
| Issa | 2022 | Random | 4 | 138 | MSCs | 1×10^6^-1×10^8^ | 3 | Yes | Cochrane Collaboration’s ROB2 tool |
| Jeyaraman | 2022 | Random | 21 | 936 | MSCs | 3.9×10^6^-1.5×10^8^ | 4 | Yes | Cochrane Collaboration’s ROB2 tool |
| Long | 2022 | Random | 28 | 1494 | MSCs | 2.6×10^5^-1.5×10^8^ | 10 | Yes | Cochrane Collaboration’s ROB2 tool |
