# Supplementary material for: A systematic review, umbrella review, and quality assessment on clinical translation of stem cell therapy for knee osteoarthritis: Are we there yet?
Source: Stem Cell Res Ther. 2023 Apr 15;14:91. doi: 10.1186/s13287-023-03332-5 (PMC10105961; doi:10.1186/s13287-023-03332-5)
Supplement: Supplementary file 2 — Additional file 2: Appendix 2. Quality appraisal checklist for case series studies and instructions for use. [file 13287_2023_3332_MOESM2_ESM.docx]

**
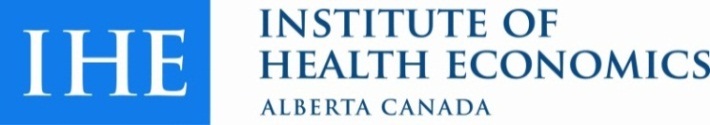
**

**Quality Appraisal Checklist for Case Series Studies
and Instructions for Use***

| **Study objective** | |
| --- | --- |
| **1.** | **Was the hypothesis/aim/objective of the study clearly stated?**  **Yes**: The hypothesis/aim/objective of the study was clearly reported (includes patients, intervention and outcome).  **Partial**: Only one or two components (patients, intervention, or outcome) were included.  **No**: The hypothesis/aim/objective was not reported. |
| **Study design** | |
| **2.** | **Was the study conducted prospectively?**  **Yes:** It was clearly stated that the study was conducted prospectively.  **Unclear**: Unclear or no information was provided.  **No:** The study clearly stated it was a retrospective study. |
| **3.** | **Were the cases collected in more than one centre?**  **Yes**: Cases were collected in more than one centre (multicentre study).  **Unclear**: Unclear where the patients came from.  **No**: Cases were collected from one centre. |
| **4.** | **Were patients recruited consecutively?**  **Yes**: There was a clear statement or it was clear from the context that the patients were recruited consecutively; or the study stated that all eligible patients were recruited.  **Unclear**: No information was provided about the method used to recruit patients in the study.  **No**: The study clearly stated that patients were not recruited consecutively; or the patients were recruited based on other criteria such as access to intervention determined by the distance or availability of resources. |
| **Study population** | |
| **5.** | **Were the characteristics of the patients included in the study described?**  **Yes**: All of the most relevant characteristics of the patients were reported (for example, number, age, gender, ethnicity, severity of disease/condition, comorbidity, or etiology).  **Partial**: Some, but not all, of the most relevant characteristics were reported.  **No**: Only the number of patients was reported.  *Note: Assessor(s) should decide which aspects are important before using the checklist.* |
| **6.** | **Were the eligibility criteria (i.e. inclusion and exclusion criteria) for entry into the study clearly stated?**  **Yes**: Both inclusion and exclusion criteria were reported.  **Partial**: Either the inclusion or exclusion criteria were reported.  **No**: Neither inclusion nor exclusion criteria were reported.  *Note: Assessor(s) should decide which aspects are important before using the checklist.* |
| **7.** | **Did patients enter the study at a similar point in the disease?**  **Yes**: It was clear from the baseline data presented in the study (for example, tables of patients’ characterises) that the majority (at least 80%) of patients entered the study at a similar point in terms of the duration and severity of the disease/condition and the presence of co-morbidities/complications.  **Unclear**: There was no baseline information on patients’ characteristics to make a judgment.  **No**: There was a wide range in the severity of the disease/condition and co-morbidities/complications in patients at baseline.  *Note: Assessor(s) should decide which aspects are important before using the checklist.* *It might be useful to discuss with specialists to determine the most important aspects that should be considered.* |
| **Intervention and co-intervention** | |
| **8.** | **Was the intervention of interest clearly described?**  **Yes**: All of the most relevant characteristics of the intervention were reported (for example, dosage, frequency or duration of intervention, administration methods, technical parameters, or characteristics of a device).  **Partial**: Some, but not all, of the most relevant characteristics were reported.  **No**: Only the name of the intervention was reported.  *Note: Assessor(s) should decide which aspects are important before using the checklist.* |
| **9.** | **Were additional interventions (co-interventions) clearly described?**  **Yes**: All of the most relevant characteristics of the co-intervention(s) were reported (for example, different type, dosage, frequency of administration, or duration); or the study clearly stated that a co-intervention was not administered for clinical reasons.  **Partial**: Some, but not all, of the most relevant characteristics of the co-intervention were reported.  **No**: No information about co-intervention(s) was provided; or only the name(s) of the co-intervention(s) were mentioned.  *Note: Assessor(s) should decide which aspects are important before using the checklist.* |
| **Outcome measures** | |
| **10.** | **Were relevant outcome measures established a priori?**  **Yes**: All relevant outcome measures were stated in the introduction or methods section.  **Partial**: Some, but not all, of the relevant outcome measures were stated in the introduction or method section.  **No**: None of the relevant outcome measures were stated in the introduction or method section. |
| **11.** | **Were outcome assessors blinded to the intervention that patients received?**  **Yes**: The relevant outcomes were assessed by individuals who were not aware of the intervention. Answer yes when blinding is not applicable or is unnecessary (for example, mortality).  **Unclear**: The study did not report whether the outcome assessors were aware of the intervention.  **No**: It was clearly stated or obvious from the context that the relevant outcomes were analyzed by individuals who were aware of the intervention provided to patients. |
| **12.** | **Were the relevant outcomes measured using appropriate objective/subjective methods?**  **Yes**: All relevant outcomes were measured with appropriate methods. These measures can be objective (for example, gold standard tests or standardized clinical tests), subjective (for example, self-administered questionnaires, standardized forms, or patient symptoms interview forms), or both.  **Partial**: Some, but not all, relevant outcomes were measured with appropriate methods.  **No**: The methods used to measure the relevant outcomes were inappropriate.  *Note: Assessor(s) should decide which methods are appropriate before using the checklist.* |
| **13.** | **Were the relevant outcome measures made before and after the intervention?**  **Yes**: The relevant outcome measures were made pre- and post-intervention; or the baseline measurements were not possible (for example, death).  **Unclear**: The study did not report when the outcome measures were made.  **No**: The outcome measures were only made post-intervention. |
| **Statistical analysis** | |
| **14.** | **Were the statistical tests used to assess the relevant outcomes appropriate?**  **Yes**: The statistical tests were used appropriately (for example, parametric test for normally distributed population vs. nonparametric test for non-Gaussian population). Answer yes if no statistical analysis was performed and reasons for this were stated.  **Unclear**: The statistical tests were not described in the methods section of the study.  **No**: The statistical tests used were inappropriate.  *Note:* *Assessor(s) should decide which statistical tests are appropriate before using the checklist. Seek expert assistance if necessary.* |
| **Results and conclusions** | |
| **15.** | **Was follow-up long enough for important events and outcomes to occur?**  **Yes**: It was clear from the information provided that the follow-up period was long enough for the majority (at least 80%) of patients, to allow for important events and outcomes (for example, changes in clinical status, adverse events) to occur.  **Unclear**: The length of follow-up was not clearly reported.  **No**: It is clear from the information provided that the follow-up period was not long enough to allow for important events and outcomes to occur.  *Note:* *Assessor(s) should define the appropriate duration of follow-up for each outcome of interest (for example, short-term and long-term adverse events).* |
| **16.** | **Were losses to follow-up reported?**  **Yes**: The number or proportion of patients lost to follow-up was clearly reported; the authors reported outcome results on all patients initially included; or the number lost to follow-up can be subtracted from the number of patients enrolled and the number of patients included in the final analysis.  **Unclear**: There was a discrepancy between the number or proportion of patients reported in tables, figures, and text.  **No**: The number or proportion of patients lost to follow-up was not reported. |
| **17.** | **Did the study provided estimates of random variability in the data analysis of relevant outcomes?**  **Yes**: The estimates of the random variability (for example, standard error, standard deviation, confidence interval for normally distributed data or range and interquartile range for non-normally distributed data) were reported for all of the relevant outcomes or could be calculated from the raw data presented in the study.  **Partial**: The estimates of the random variability were reported for some, but not all of the relevant outcomes.  **No**: The estimates of the random variability were not reported for any of the relevant outcomes. |
| **18.** | **Were the adverse events reported?**  **Yes**: The undesirable or unwanted events during the study period or within a pre-specified time period were reported; or the absence of adverse event(s) was mentioned in the study.  **Partial**: Some, but not all, important adverse events were reported.  **No**: There was no statement about the presence or absence of adverse events.  *Note:* *Assessor(s) should decide which adverse events are most important. Seek clinical expert assistance if necessary.* |
| **19.** | **Were the conclusions of the study supported by the results?**  **Yes**: The conclusions of the study were supported by the evidence presented in the results and discussion sections.  **Unclear**: Unclear conclusion statement that makes it difficult to link the presented evidence to conclusions.  **No**: The conclusions were not supported by the evidence presented in the results and discussion sections. |
| **Competing interests and sources of support** | |
| **20.** | **Were both competing interests and sources of support for the study reported?**  **Yes**: Both competing interests and sources of support (financial or other) received for the study were reported; or the absence of any competing interest and source of support was acknowledged.  **Partial**: Either the competing interest or source of support was reported.  **No**: Neither competing interests nor sources of support were reported. |

### *Considerations when using the checklist:

- Some of the criteria may not be applicable for some interventions/conditions; therefore our recommendation to users is to review the checklist and include only those applicable, before beginning the appraisal.
- To reduce disagreements, reviewers should decide about the important aspects to be considered for some of the criteria (such as characteristics of the patients included in the study, or description of the intervention), prior to beginning the appraisal.

**This checklist should be cited as:**

Institute of Health Economics (IHE). Quality Appraisal of Case Series Studies Checklist. Edmonton (AB): Institute of Health Economics; 2014. Available from: <http://www.ihe.ca/research-programs/rmd/cssqac/cssqac-about>

**References**

Moga C, Guo B, Schopflocher D, Harstall C. *Development of a quality appraisal tool for case series studies using a modified Delphi technique*. Edmonton: Institute of Health Economics; 2012. Available at <http://www.ihe.ca/advanced-search/development-of-a-quality-appraisal-tool-for-case-series-studies-using-a-modified-delphi-technique> . Accessed January 8, 2016.

Guo B, Moga C, Harstall C, Schopflocher D. A principal component analysis is conducted for case series quality appraisal checklist. *Journal of Clinical Epidemiology* 2016;69:199-207.
